# Pollination ecology of the ghost orchid (*Dendrophylax lindenii*): A first description with new hypotheses for Darwin’s orchids

**DOI:** 10.1038/s41598-019-49387-4

**Published:** 2019-09-06

**Authors:** Peter R. Houlihan, Mac Stone, Shawn E. Clem, Mike Owen, Thomas C. Emmel

**Affiliations:** 1 McGuire Center for Lepidoptera and Biodiversity, Florida Museum of Natural History, University of Florida, Gainesville, FL 32611 USA; 2 Department of Biology, University of Florida, Gainesville, FL 32611 USA; 3 International League of Conservation Photographers (iLCP), Washington D.C., USA; 4 Corkscrew Swamp Sanctuary, Audubon Florida, Naples, FL USA; 5 Fakahatchee Strand Preserve State Park, Copeland, FL USA; 6 Florida Biodiversity Foundation, Gainesville, FL USA

**Keywords:** Conservation biology, Community ecology, Tropical ecology, Entomology, Plant ecology

## Abstract

The structural variation of orchids enables myriad fascinating symbiotic relationships with organisms across kingdoms. Orchids are frequently known for having elaborate arms races with their pollinators that result in intricate morphologies in both parties, and flowers with long corollas hypothesized to be pollinated only by individual species of long tongued hawkmoths are of particular concern for conservation. Florida’s endangered ghost orchid, *Dendrophylax lindenii*, has long been confidently assumed to be pollinated by one species (*Cocytius antaeus*), despite the presence of a resident community of multiple suitable long-tongued candidates. Here we present the first description of ghost orchid pollination, and describe novel remote camera trapping methods. Pollination of *D. lindenii* by *Pachylia ficus* disproves long-standing hypotheses concerning the pollination ecology of long-spurred orchids, and new multiple pollinator hypotheses are proposed. We discuss the broader implications for the conservation of an endangered species, orchids globally, and the importance of Everglades restoration.

## Introduction

Charles Darwin first discussed the evolutionary relationships between insect pollinators and plants in the Origin of Species^1^. Elaborating on this concept of coevolution in “The Fertilisation of Orchids”, Darwin presented the case of a long-spurred orchid in Madagascar, *Angraecum sesquipedale* (Vandeae: Angraecinae), which he predicted was pollinated by a long-tongued hawkmoth^2,3^. The structural variation of orchids enables myriad fascinating symbiotic relationships with organisms across the fungi, plant, and animal kingdoms^4^. Orchids are frequently known for having elaborate arms races with their pollinators that result in intricate morphologies in both parties^5,6^. Due to their specialized habitat preferences and occasionally low abundance in the wild, orchids are of particular interest in climate change research and of conservation concern^7–9^. Yet considering the immense diversity of species, detailed species-level understandings of orchid natural history remain limited, and oftentimes pollination syndromes are the only hypotheses available from which to predict candidate species or guilds of potential pollinators. Tragically, the majority of the world’s orchids are threatened by habitat degradation and poaching^9^. As a result of their complex interactions with pollinators, orchid declines are likely to seriously impact populations of insects^8^, most notably including moths specialized for orchid pollination^10,11^. Thus, orchids with long corollas that are hypothesized to be pollinated by individual species of long-tongued hawkmoths are of particular concern^12,13^.

Within the predominantly African Angraecinae subtribe, two genera radiated into the Americas, *Dendrophylax* and *Campylocentrum*^14,15^. The ghost orchids (*Dendrophylax spp*.) range from south Florida throughout the Caribbean and many species are restricted to single islands^14,16^. These endemic species exhibit morphological adaptations to island-specific ecological conditions^17^, while numerous species maintain a long nectar spur throughout the genus. Of the fifteen described species, zero have definitively described pollinators, and many are threatened with extinction.

Found in south Florida and Cuba, *Dendrophlax lindenii* is one of the most well-known orchids in the world, in part due to popular media such as the novel, The Orchid Thief^18^, and film, *Adaptation*, which showcased the poaching of this endangered species. Despite widespread attention, many factors have hindered scientific research to understand the natural history of *D. lindenii*, confounding the quest to understand the species’ pollination ecology; some factors include: the species’ rarity, restricted access to areas where it occurs, the remarkably strenuous conditions posed for field researchers in these habitats, historical political relations between the United States and Cuba, the temporally nocturnal emission of volatile compounds^19^, and the need for advanced camera technology.

Due to the parallel nature to Darwin’s orchid-hawkmoth system in Madagascar^2^, it has long been hypothesized that long-spurred Caribbean angraecoids like *D. lindenii* are pollinated by long-tongued hawkmoths^13,20^. Creamy white in color, vespertine, and possessing a long nectary that can vary from 12–16 cm, *D. lindenii* is sphingophilous as defined in Haber & Frankie^21^. Although a single hawkmoth (Lepidoptera: Sphingidae) has long been hypothesized as the sole pollinator of *D. lindenii*, the pollination ecology and phylogeography of the entire genus remain poorly understood (pers. comm. Mark Whitten & Norris Williams). Without definitive confirmation, widespread conjecture within the orchid community has long stated with confidence that only one hawkmoth species in Florida, *Cocytius antaeus* (giant sphinx moth), fits the morphological description of having a proboscis of comparable length to the corolla of *D. lindenii*, and thus must be the only pollinator^22^. However, contrary to the assumption of this long proliferated “just so story”^23^, numerous species of Sphingidae, and other Lepidopterans, possess a proboscis length that would be sufficient to reach the nectar reward of *D. lindenii* in south Florida, and no reports have acknowledged the presence of closely related hawkmoths possessing comparable proboscis lengths, notably the *Amphonyx* and *Manduca* genera, other species of *Cocytius* in Cuba, or even longer still, one species occurring in Cuba with a proboscis length more than twice that of *C. antaeus, Neococytius cleutenius*^24^.

Ghost orchid (*Dendrophylax lindenii*) studies thus far have investigated mycorrhizal relationships^22,25^, host tree affinities^26,27^, micropropagation^28^ d, and volatile compound composition^19^. Despite much allure to the pollination story of this charismatic species, little is actually known about the ecological interactions between insects and ghost orchids^29^. These gaps are problematic for efforts surrounding the future conservation of orchids, which face a global decline^30^. With ongoing alterations to natural habitats, and climatic shifts, ecological data describing the pollination of endangered species are critical for establishing more effective conservation measures, particularly considering the sensitivity of south Florida’s plant communities to perturbations in hydrology and fire regimes with changes in land use and management^31^ and in light of predictions of increased frequency of extreme weather events^32^. Here we present novel camera trapping methods developed to document the pollination of *D. lindenii*, we report on successful findings, and urge for these approaches to be utilized more widely for similarly threatened and data deficient species.

## Materials and Methods

### Fieldwork

Individuals of *D. lindenii* were located, observed, and monitored from June 2014 through July 2017 in the Fakahatchee Strand. During this time, 14 visits were made to this study site, totaling 47 days in the field and amounting to 423 hours searching for and observing the orchids. A total of 29 nights was spent light trapping to attract insect pollinators and inspect hawkmoth proboscides for orchid pollinia. The final camera trapping season of 2018, from which the results here were recorded, utilized novel methods developed and custom-built by MS, was carried out by MS & PRH in Corkscrew Swamp Sanctuary, informed by the culmination of natural history information amassed in previous years. These efforts were concentrated on the “super ghost” at Corkscrew Swamp Sanctuary, a cluster of root masses from at least three separate individuals stacked on top of one another, situated approximately 15 m up on a cypress tree that can be viewed by visitors through a spotting scope or binoculars at a distance of approximately 75 m from a boardwalk.

### Study sites

Corkscrew Swamp Sanctuary: Corkscrew Swamp Sanctuary (centered ca. 26°23′60″N, 81°36′37″W) was established by National Audubon Society in 1954 to protect a 5,600 acre tract of old-growth *Taxodium distichum* (bald cypress) forest and its associated plants and wildlife from logging. Currently 13,400 acres, the sanctuary’s central bald cypress swamp contains trees exceeding 500 years old^33,34^ and is surrounded by a mosaic of freshwater marsh, wet prairie, pine flatwoods and hardwood forest. A recent floristic inventory of the Sanctuary documented 773 infrageneric taxa of vascular plants, including 29 listed as endangered or threatened in Florida^35^.

Fakahatchee Strand Preserve State Park: The Fakahatchee Strand (centered c. 26°00′00″N 81°25′01″W) is a unique sub-tropical forest, characterized by seasonally flooded sloughs, beneath a canopy of *T. distichum*, that are dominated by *Fraxinus caroliniana* and *Annona glabra*, the primary hosts in Florida for the epiphytic *D. lindenii*. Old growth cypress in the Fakahatchee Strand was heavily logged in the early 20^th^ Century. The presence of Roystonea regia (royal palm) in cypress forest adds to their distinctiveness, as this sympatry is not found anywhere else in the world. Fakahatchee contains the highest diversity of orchids (49) and bromeliads (14) in the United States (MO, unpubl. data).

### Preliminary studies

Multiple methods were employed, tested, and adapted over five flowering seasons in order to attempt recording visitations to *D. lindenii*. During the course of this study, camera trapping technology progressed immensely, enabling remarkable precision of smaller subjects, including moths. This progression and implementation is noted here, with the primary focus on novel methods that yielded results during the 2018 field season.

In 2014, one HD Infrared Night Vision Camcorder was deployed on a new bloom of *D. lindenii* in Fakahatchee to record HD video from sunset to sunrise nightly for two weeks. Two Bushnell Trail Cameras were also deployed on orchids within the same slough. In 2016 and 2017, a Canon XF205 HD infrared camera was deployed on a tripod to film one individual with two flowers for ten hours overnight on 20 separate nights, totaling 200 hours of camera monitoring. A Trail Master TM 1550 BAT Active infrared trigger for a Canon 7D Mark II SLR was also deployed in for 5 nights in July 2017. From 2014–2017, PRH spent a total 23 full nights, from sunset to sunrise, seated atop a 2.5 m ladder in flooded sloughs to trigger a Canon 7D Mark II SLR mounted on a tripod using a Canon Remote Controller TC-80N3 and Remote Shutter Extension cable to position 4 m from the camera. All described methods proving unsuccessful, custom remote technologies were engineered as follows.

### Novel camera trapping development & deployment

In June 2018, efforts were concentrated at Corkscrew Swamp Sanctuary on a cluster of root masses from at least three separate individuals stacked on top of one another, discovered in July 2007 and locally termed the “super ghost”. As multi-flower orchids have been shown to yield higher fruit set^36^, this concentration of flowers provided the highest perceived probability of capturing pollinators. For this reason, it was selected to deploy a camera trap.

**Figure 1:**
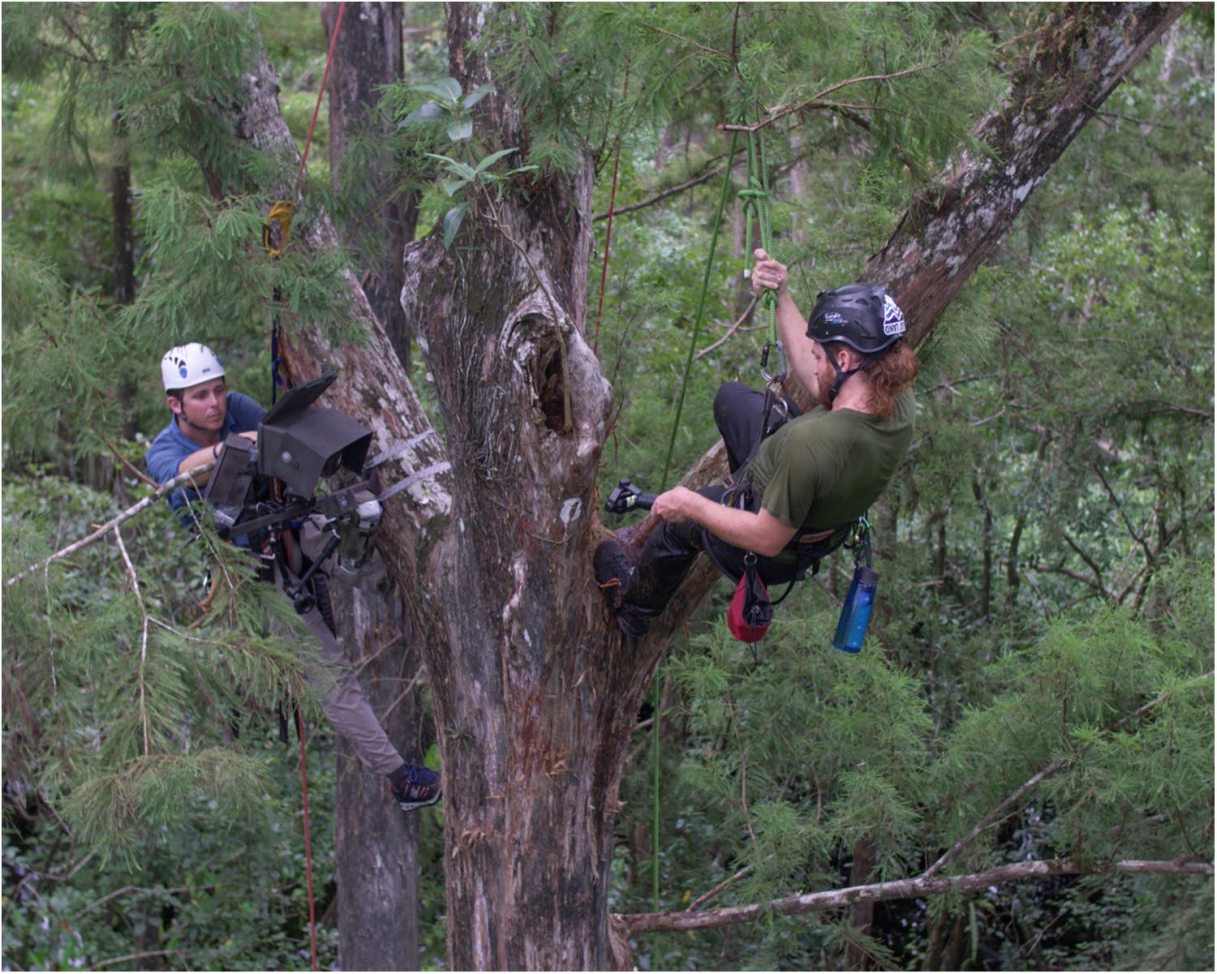
Authors MS (left) and PRH (right) climb an old growth bald cypress at Audubon’s Corkscrew Swamp Sanctuary to install a custom remote camera trap for recording visitors to D. lindenii, situated on the central trunk. Drone photo courtesy of Grizzly Creek Films.

Situated at 15 m above the flooded forest, the highest of any known *D. lindenii*^35^, the site was accessed by tree climbing. A 0.34 kg throw weight attached to a 50 m throw line was launched, using a Notch Big Shot weight launcher, over a branch at 20 m height. A 50 m long tachyon 11.5 mm static climbing rope was then hoisted with a cambium saver. This rope was climbed on the right side of the orchid to then install a second rope that would be utilized to access the orchid from the left side and install the camera trap (Fig. 1).

A Canon 1200D DSLR camera with a 10–20 mm Sigma lens and two Nikon SB-28 flashes was first deployed on June 27, 2018. In recording only one image of the first visitation, the Canon 1200D (3 frames per second) was replaced with a faster Canon 7D Mark II (10 fps) on July 17, 2018. Installed at a height of 15 m on *T. distichum*, MS designed a custom steel arm, built to hold over 23 kg, mounting all elements of the entire camera trap system (camera, flashes, and passive infrared trigger). This prototype, named the “TreePod”, consisted of a square steel tube 66 cm long welded to a 6.4 mm steel plate with support brackets, forming a T-shape at one end. Two steel U-bolts were affixed on each side of the support brackets. The steel arm was positioned perpendicular to the tree and held in place by load-bearing ratchet straps that could be threaded through the U-bolts to hold the arm in place and secured to the tree.

A Camtraptions PIR sensor, a passive infrared triggering system, was deployed to monitor the three dimensional multi-flower zone. Passive infrared works to detect subtle changes in infrared light, through movement or heat from a body passing in front of the sensor. The wide cone of detection on the Camtraptions PIR sensor allowed monitoring to encompass all flowers as potential locations for visiting moths, without having to guess or choose a single focal point.

Regular maintenance visits to the trap were conducted to adjust the angle of the camera and the PIR sensor, depending on the location of new blooms, and to replace batteries. The last frame the camera recorded during the season was September 9, 2018. The camera battery died that afternoon and no further images were recorded.

### Light trapping

To attract hawkmoths for pollinia inspection, a combination of mercury vapor lamps, metal halide bulbs, and black lights were utilized at eight sites on 40 nights from 2014 to 2017 along Janes Scenic Drive between the Fakahatchee Strand Preserve State Park Headquarters and the Picayune Strand State Forest, and for two nights in Corkscrew Swamp Sanctuary in August 2018. Moths were also searched for opportunistically at night, detected by their eye shine with headlamps or captured in flight by hand nets.

### Proboscis measurements

To demonstrate the presence of a diverse community of long-tongued hawkmoths that occur within the distribution of *D. lindenii*, specimens were measured in the collections at the McGuire Center for Lepidoptera & Biodiversity at the Florida Museum of Natural History. Due to the difficultly of relaxing the proboscis on dried, prepared specimens, individuals were selected where the proboscis was already partially or fully exposed (as opposed to hidden between the mouthparts). Representative specimens were measured for eleven species. Specimens were selected from Florida or the southeastern US for regional relevance and because intraspecies proboscis length can increase where distribution extends further into the tropics^29^, which is evident for many species here that also occur in Central and South America^37^; thus these are conservative records for these species as the objective was to demonstrate that additional sphingid species exist that fit various tongue length hypotheses, which have not been given consideration previously. A paintbrush was utilized to coat probosces with heated potassium hydroxide (KOH), without detaching the proboscis, rendering them flexible enough to manipulate and uncoil.

## Results

In 2018, the camera trap was active for 75 days, triggering a total of 7,938 images, recording 23 images capturing visitations by two species of hawkmoths, *Cocytius anteaus* and *Pachylia ficus*. Due to the wide cone monitored by the PIR sensor, there were many instances of false triggers from wind moving the ghost orchids.

**Figure 2:**
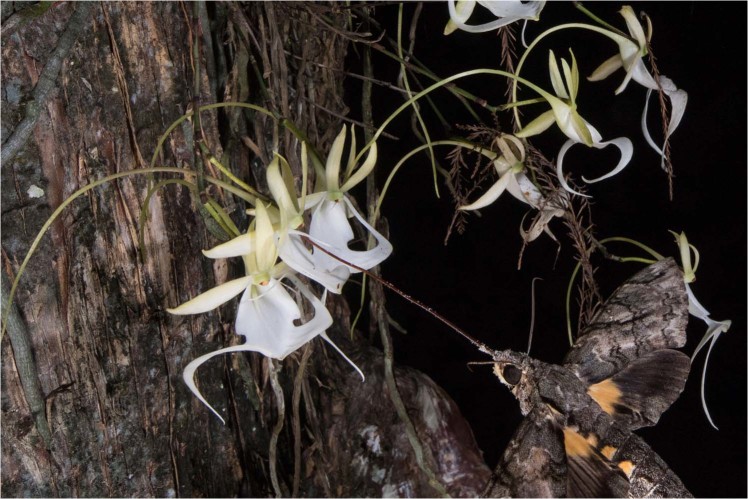
*Cocytius antaeus* feeds from a ghost orchid at a distance from the flower. Pollen from Ipomoea alba is present on the moth’s head. Photo by Mac Stone.

**Figure 3:**
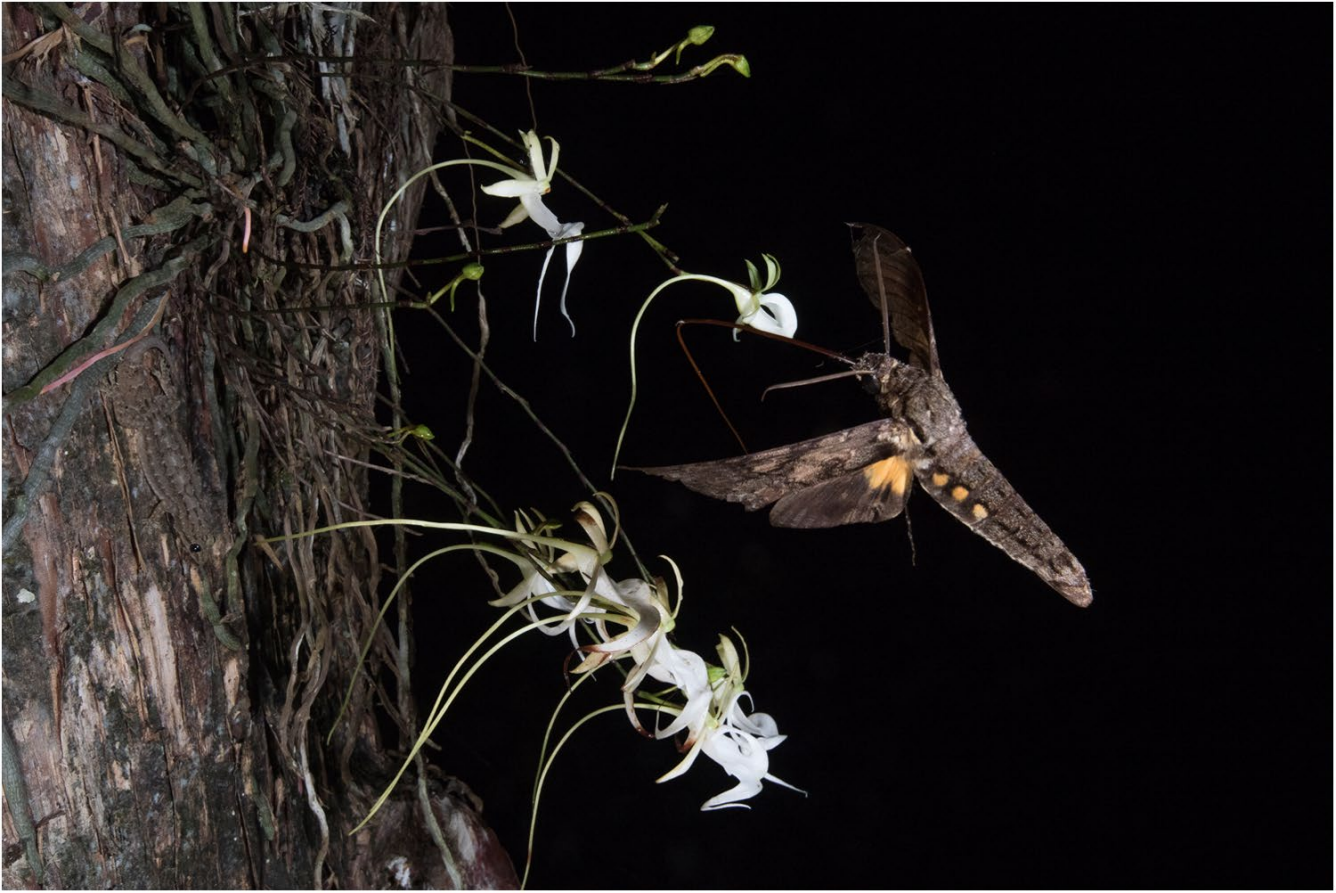
*Cocytius antaeus* visits the “super ghost” cluster at Corkscrew Swamp Sanctuary, where a gecko awaits on the bald cypress trunk amongst the root mass of *D. lindenii*. Photo by Mac Stone.

*Cocytius antaeus* individuals were recorded (Fig. 2) on three separate dates: July 15, 2018, August 23, 2018, and September 8, 2018. On the first date in July, two images were captured (22:16:39 hr), and on the last date in September, one image was recorded (02:31:09 hr). On August 23^rd^, an individual of *C. antaeus* was recorded over the course of two minutes, with images captured at 23:15:15 hr (3), 23:15:46 hr (3), and 11:17:15 hr (3). On this occasion, a gecko was present on the trunk amongst the root mass (Fig. 3), which the moth approached and made contact using its proboscis. All individuals were males, and images of the visitation spanning 120 seconds appear to be of the same individual. Pollinia was not affixed to the moth on any of these three visitations, however, pollen from *Ipomoea alba* was visibly covering the head of the final two individuals. Pollinia did not appear to be extracted from *D. lindenii* after visiting either.

**Figure 4:**
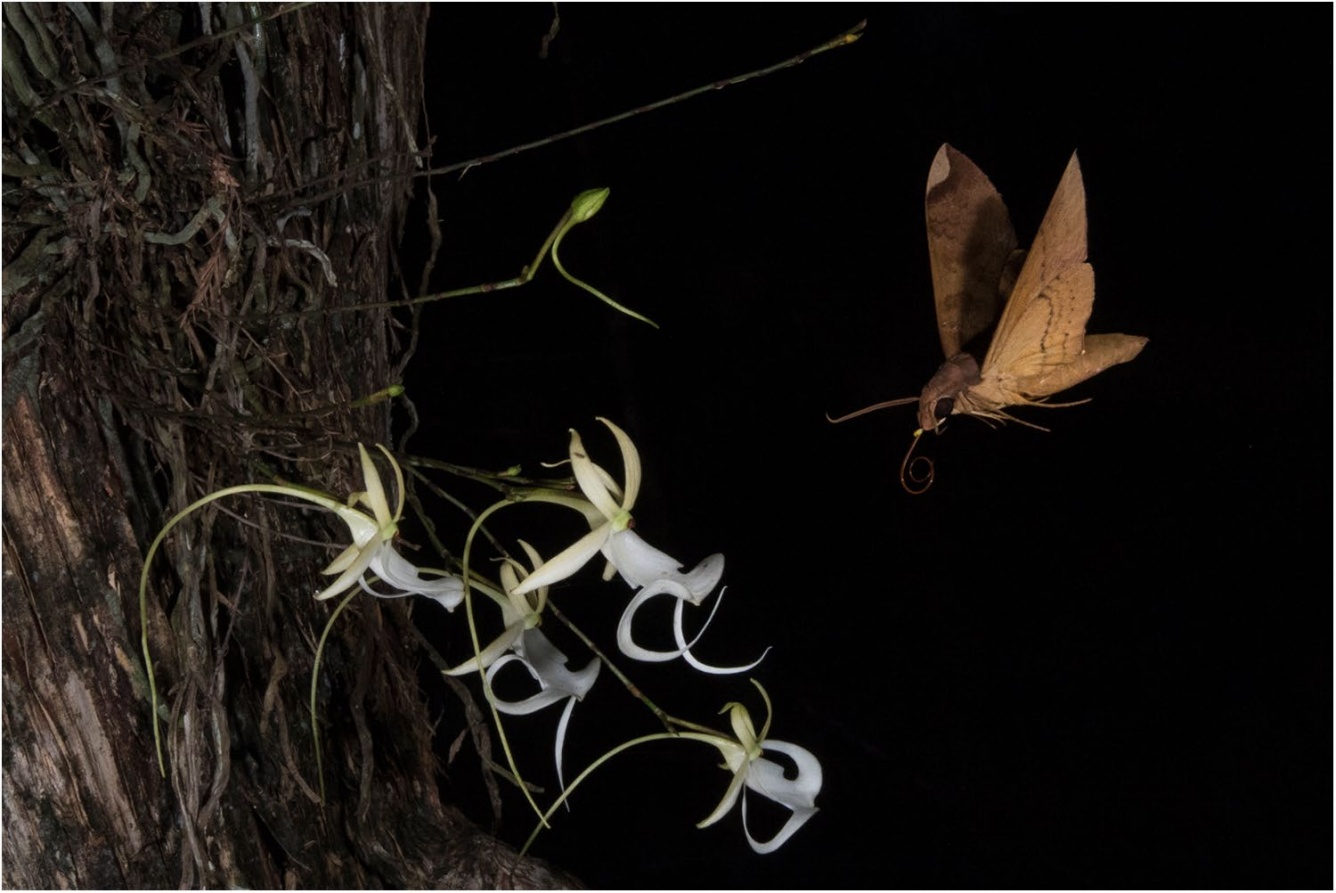
*Pachylia ficus* visits a cluster of ghost orchids with pollinia from *D. lindenii* affixed to the base of its proboscis near its head. Photo by Mac Stone.

**Figure 5:**
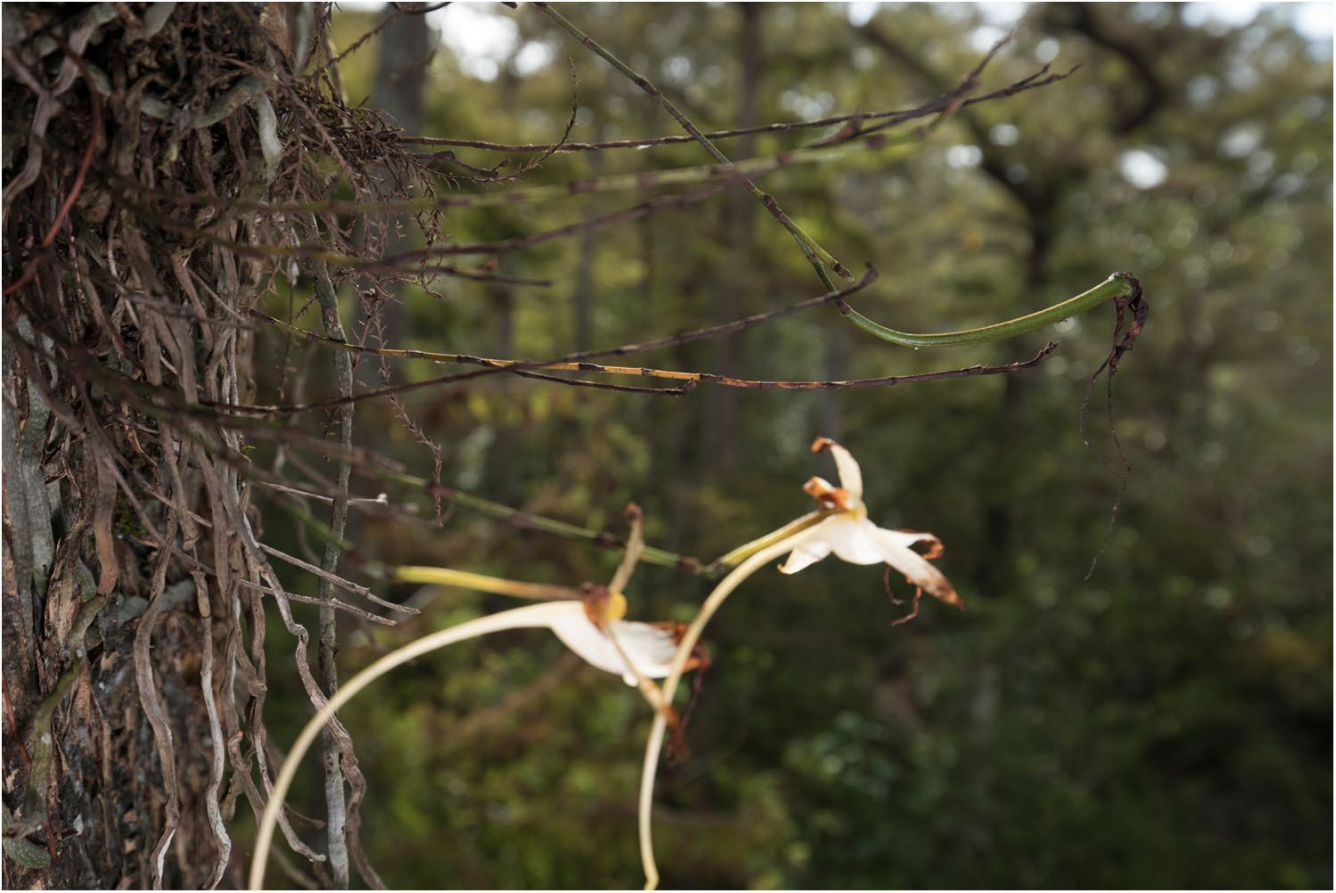
A seed pod produced during the end of the flowering season, on October 2, 2018. Photo by Mac Stone.

*Pachylia ficus* individuals were recorded on three images during two occasions on July 20, 2018 at 00:00:28 hr (2) and 06:23:50 hr (1). On the first visit, *D. lindenii* pollinia was affixed to the base of the moth’s proboscis near its head (Fig. 4). The second visitation appears to be of a separate individual based on the substantial wing wear that is unlikely to have occurred over a period of six hours. A seed pod was developing when the camera was serviced the final time on October 2, 2018 (Fig. 5).

Across this sampling period in Fakahatchee (2014–2017), zero individuals of *Cocytius antaeus* were attracted to light traps. A total 12 individuals were located either with the use of a flashlight to search for eye shine of the moths resting on vegetation, or by capturing with a hand net when observed flying along the park road. Light trapping was conducted on two evenings in Corkscrew Swamp, for a shorter period of time (2 hrs), first at the edge of a meadow with a mercury vapor lamp, which attracted a male *C. antaeus* and a subsequent night with a blacklight at 30 m on *T. distichum* where a male *C. antaeus* was spotted previously at 20 m while tree climbing.

**Figure 6:**
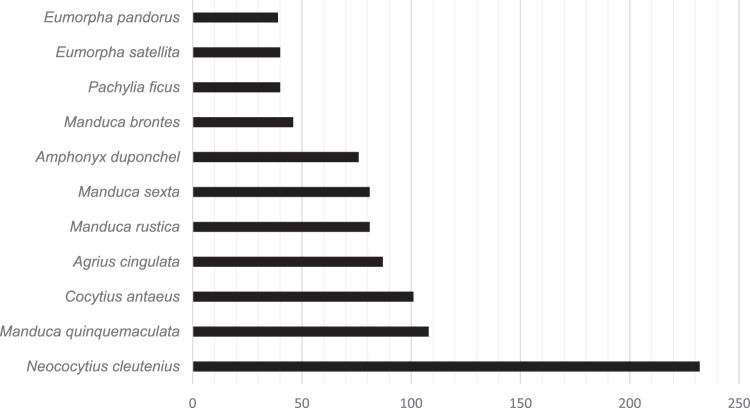
Proboscis length (mm) of species of long-tongued hawkmoths in south Florida and Cuba, measured in the collections at the McGuire Center for Lepidoptera & Biodiversity at the Florida Museum of Natural History, demonstrate that multiple species occur in the distribution of D. lindenii that possess longer proboscides than the long hypothesized sole pollinator of C. antaeus, and given the confirmed pollination by P. ficus, new hypotheses should consider a much larger community of hawkmoths with equal or greater proboscides.

Proboscis lengths were measured for a total of eleven hawkmoth species (Fig. 6) possessing lengths comparable to *P. ficus* (40 mm) and longer (Fig. 6), including *Eumorpha satellita* (39 mm), *E. pandorus* (39 mm), *Manduca brontes* (46 mm), *M. sexta* (81 mm), *M. rustica* (81 mm), *Agrius cingulata* (87 mm) *Amphonyx duponchel* (76 mm). Notably, specimens of *M. quinquemaculata* (108 mm) and *Neococytius cleutenius* (232 mm) possessed longer probocides than *C. antaeus* (101 mm). Several additional species of interest (e.g. *Amphonyx vitrinus, Adhemarius daphne, Cocytius haxairei, Dolba hyloeus*) were either not located in the collections, or were not prepared due to the fragility of the specimen.

## Discussion

Pollination syndromes have been utilized to hypothesize guilds of pollinators since Darwin^2^. While direct observation of nocturnal pollination can be difficult for a multitude of reasons, floral morphology and resident pollinator communities inform our understanding of likely interaction scenarios. These hypotheses are especially beneficial for rare and/or endangered flowers, and their conservation; in these cases, any information regarding interactions between insects and plants is uniquely valuable. However, until data exist to confirm pollination they should be treated as hypotheses rather than fact.

Previous tongue length hypotheses for *D. lindenii* were misleading due to the placement of pollinia on the orchid. A proboscis of equal or greater length to that of the nectar spur would allow a visitor to extract all of the nectar, but may be capable of doing so without coming into contact with the flower. Curvature of a flower’s corolla has been shown to improve nectar discovery in *Manduca sexta*^38^, which may guide Lepidopterans toward the pollinia of *D. lindenii*. It appears that hawkmoths with probocides of a length slightly shorter than that of the nectar spur are encouraged to dive toward the flower to fully penetrate its proboscis to maximize nectar retrieval, increasing the probability of pollinia affixing to the moth and being deposited on a subsequent flower. Nectar volume and height within the corolla is an additional factor that may influence the minimum proboscis length necessary, and could also further distance moths with long probocides from the pollinia. Therefore, any hawkmoth containing a proboscis long enough to surpass the curvature of *D. lindenii’s* corolla may obtain the minimum volume of nectar necessary to entice it further into the flower, leading to the extraction and deposition of pollinia.

Lepidopterans with substantial proboscis lengths capable of maximum nectar extraction without contacting pollinia function as nectar robbers. This tactic may be employed by *C. antaeus*, which was recorded visiting *D. lindenii* on three separate occasions throughout the flowering season, without extracting pollinia. In one instance (Fig. 2), an individual *C. antaeus* is seen feeding on *D. lindenii* from a distance while covered in pollen from *Ipomoea alba*. This could also occur by other long-tongued hawkmoths in *D. lindenii’s* distribution, including *Manduca quinquemaculata*, and the extremely long-tongued *Neococytius cleutenius*. Visitors benefit from this behavior by avoiding close contact with the flower, allowing them to forage at a distance from ambush predators that frequently sit and wait at sphingophilous flowers^39^, including geckos (Fig. 4; unpubl. data, Borneo), frogs, toads (PRH, pers. observ., Borneo, Madagascar, French Guiana), and spiders^29,40^.

It is widely speculated that long-spurred orchids have lengthy corollas to eliminate loss of pollinia to generalist species that are unlikely to visit another individual of the same species, wasting the intensive energetic investment of flower production^11^. The volatile compounds emitted from hawkmoth-pollinated flowers are attractive to many nocturnal Lepidoptera, possibly from great distances, with visual cues employed to hone in on flowers at closer proximity^41^. Unlike Darwin’s extreme hawkmoth system concerning *Angraecum sesquipedale* and *Xanthopan morganii* in Madagascar, the nectar spur of *D. lindenii* is far shorter, and falls within a spectrum of proboscis lengths possessed by resident pollinators in Florida and Cuba. Results here demonstrate that hawkmoths with a proboscis length much shorter than *C. antaeus* are capable of pollinating *D. lindenii* which provides support for a multiple pollinator community hypothesis consisting of a diverse guild of moderate to long tongued hawkmoths; such communities have been shown to partition resources temporally^42^, and timestamped camera trap images can be utilized to investigate these behavioral patterns. The potential of other Lepidopterans should be considered now as well. Pollination here by *P. ficus* indicates that *C. antaeus*, the long suspected pollinator, is not the only candidate, possibly not even a primary one, and actually may be robbing nectar at the orchid’s detriment. With the low population number of *D. lindenii* today, combined with a high extinction rate for relatively recently colonized and diversified orchids^43^, a multiple pollinator community may provide the best survival strategy for the species. Given the proboscis length of *P. ficus*, far shorter than the hypothesized pollinator, many more hawkmoth species with equal or greater proboscis lengths (Fig. 6) should be considered as potential candidates.

Lepidoptera species richness and abundance tend to be relatively low in swamp forests^44–46^, which holds true for the Everglades Basin of south Florida. Accordingly, any interactions within a Lepidoptera depauperate community on rare and endangered flower species are of importance to the understanding of their natural history. With more than 49 species of orchids in Fakahatchee alone, the highest diversity in the United States^47^, only three species of Angiosperms altogether are thought to be pollinated here at night by hawkmoths: *D. lindenii* (Orchidaceae), *Crinum americanum* (Amaryllidaceae), and *Ipomoea alba* (Convolvulaceae). Given the extreme scarcity of *D. lindenii* and relatively great abundance of *I. alba*, shown pollinated by *C. antaeus* here, and *C. americanum* pollinated by *Dolba hyloeus* in Houlihan^29^, it is plausible that these latter two non-orchid sphingophilous flowers may be important nectar sources in sustaining hawkmoth communities, in order to remain resident to pollinate *D. lindenii*. While historical abundance was higher throughout the species’ Florida distribution, today *D. lindenii* may be dependent the presence of other sphingophilous flowers to increase its own pollination success, indicating a reality in which coevolutionary single species relationships between one hawkmoth and one orchid, ideal for maximizing fidelity, would no longer be advantageous.

Florida and Cuba populations of *D. lindenii* share numerous species within their hawkmoth communities, with 50 species of Sphingidae found in Florida’s Everglades Basin^48^, and 60 in Cuba^49^, and occasional vagrants to both. Despite the likelihood of Caribbean hawkmoth migrations (pers. comm. Dan Janzen & Winnie Hallwachs), and the feasibility of flight between Florida and Cuba, the disjunction of flowering times between these populations of *D. lindenii*^22,50^ indicates that pollinia transfer between these populations, separated spatially and temporally, is unlikely. Consequently, the biological relevance of these two *D. lindenii* populations being considered one in the same is questioned, and morphometric and genetic analyses should be conducted.

Seasonality and climatic fluctuations influence the phenology of tropical flooded forests^51^, and in turn the abundance and composition of Lepidoptera communities^45^. A few studies have correlated hawkmoth abundance with phenology of the flowering species on which they forage^21,52,53^, and fruit set in orchids has been shown to be higher in correlation with increased flower production^36^. In the wild, *D. lindenii* can produce single or multiple flowers annually until the production of inflorescences depletes its nutrient stores (MO, unpubl. data), after which it is common for an individual to undergo dormancy until these nutrients have been replenished^28^. Collectively, Corkscrew’s “super ghost” is unique in that it ranks among the largest ghost orchid root masses known and it flowers more prolifically than others in south Florida, having produced more than 40 inflorescences in 2014 (SEC, unpubl. data). Long term monitoring of *D. lindenii* by MO in the Fakahatchee Strand revealed that after germination in 1992, two individuals produced first flowers in July 2008 and July 2009, placing the age at first flower for these two wild *D. lindenii* at 16 and 17 years, respectively (MO, unpubl. data), while in controlled laboratory settings, time from germination to inflorescence can be expedited^28^. Additionally, in the 25-year dataset monitoring ~450 ghost orchids at Fakahatchee Strand, the largest population of *D. lindenii* in Florida, seed pod production by *D. lindenii* was found to increase in the year following intense hurricanes (MO, unpubl. data), defined as Category 3 or higher, compared to an annual seed pod production in other years between zero and two; after Hurricane Wilma made landfall over Fakahatchee Strand in October 2005, seven seed pods were produced during summer 2006, and six seed pods were produced during summer 2018 after Hurricane Irma directly impacted Fakahatchee Strand in September 2017 (MO, unpubl. data). While hurricanes have immediate destructive impacts on natural environments, including Hurricane Ivan’s devastation on the population of *D. lindenii* in western Cuba^54^, and the loss of one of Corkscrew Swamp’s individuals to Irma in 2017, replenished aquifer conditions imposed in the aftermath of intense hurricanes briefly resemble that of an era prior to the drainage of the Everglades. Further investigation is necessary to understand the correlations between these abiotic factors on *D. lindenii* flower production in the wild.

The Fakahatchee Strand was heavily logged for *T. distichum* (bald cypress) in the first half of the 20^th^ Century, effectively removing an entire upper strata from the Fakahatchee, and lowering the canopy. Today, the epiphytic orchids in Fakahatchee occur predominantly on pond apple (*Annona glabra*) and pop ash (*Fraxinus caroliniana*), which may be attributed solely to mycorrhizal relationships and host plant affinities^22^. However, it is difficult to assess what former role *T. distichum* served as a host tree due to its widespread selective logging. Corkscrew Swamp Sanctuary is home to the largest remaining old growth cypress stand in the world, where the only known *D. lindenii* are found within the virgin cypress stand, attached to *T. distichum*. Prior to cypress logging and orchid poaching, *T. distichum* may have hosted a higher proportion of the *D. lindenii* population than present day. Corkscrew Swamp sheds light on the ghost orchid’s historical natural habitat in which *T. distichum* expands the vertical stratification where *D. lindenii* can occur and also provides more surface area suited to sustain older, larger, and more fruitful epiphytic orchids. Lepidoptera community composition fluctuates greatly with respect to vertical stratification within tropical forests^44^; expanding the species’ height distribution exposes *D. lindenii* to a more diverse pollinator community, and attracts pollinators from greater distances due to the wider dispersal of volatile compounds in and above the upper canopy, increased reflectance of ambient light on floral color, and closer proximity to far ranging hawkmoths that exhibit above-canopy cruising altitudes. The epiphytic presence of *D. lindenii* in the cypress canopy also aides wind dispersal of seeds. Ghost orchid recruitment in the understory tends to occur within the immediate vicinity, often on the same tree or within the same slough as the parent plant (pers. observ., PRH & MO), with population dynamics functioning as epiphytic metapopulations as in Winkler *et al*.^55^. At lower heights in flooded forests, seeds are often dispersed into the water rather than on a potential host tree. Increasing the distance above the forest floor enables more surface area for seeds to colonize throughout the forest on their descent, and also a higher likelihood for windward travel through the upper canopy, increasing seed dispersal and genetic diversity.

The bald cypress canopy also serves as a microclimate buffer, stabilizing abiotic conditions within the forest critical to the life cycle of *D. lindenii*^28^. While widespread hydrologic disruption has been well-documented with the channelization and compartmentalization of the Florida Everglades, regional impacts of land use changes and increased groundwater extraction, concurrent with increased population growth, are becoming evident. Corkscrew Swamp Sanctuary’s 55-year hydrologic record indicates a 27% decrease in the hydroperiod of the sanctuary’s bald cypress swamp, with most change taking place 1990 to 2015^56^. While the exact cause of these changes are unknown, a combination of upstream and downstream development, increased groundwater extraction, and increased evapotranspiration are likely culprits. For species sensitive to temperature and/or humidity, particularly epiphytic orchids, these changes translate to an absence of standing water below the bald cypress canopy for nearly three additional months during the dry season, significantly increasing their vulnerability to microclimate extremes. Notably, ghost orchids were more abundant at Corkscrew Swamp Sanctuary^35^ and Fakahatchee (MO, unpubl. data) prior to abnormal freezes that decimated populations in recent years. Over-drainage of the western Everglades also places cypress swamps at risk of increasingly severe wildfires^57^. Small and large scale wetland restoration projects within the Greater Everglades can help maintain and restore climatic stability for both ghost orchids and their hawkmoth pollinators.

Today, the majority of world's orchids are threatened^30^, and many species of orchids remain data deficient, particularly with respect to pollination ecology. Understanding *in-situ* ghost orchid reproduction is imperative for enacting effective conservation, especially for *ex-situ* propagation and reintroduction efforts. Remote camera trapping methods described here provide new insight into approaches that can be implemented for identifying pollinators of orchids that do not have any documented. Interdisciplinary collaborations between researchers (entomologists and botanists), in coordination with photographers and tree climbers, are critical in addressing these complex conservation issues. Plagued with poaching, historical logging, sprawling development and habitat degradation, and climate change, ghost orchids have a fragile existence, and elevated protection status, from state to Federal, is strongly recommended.

Perhaps Darwin’s most important orchid prediction of all was what he foreshadowed of the conservation of these intricate hawkmoth-orchid systems, in which more than a century and a half ago, he predicted of the Madagascan star orchid, “If such great moths were to become extinct in Madagascar, assuredly the Angraecum would become extinct. On the other hand…the extinction of the Angraecum would…be a serious loss to these moths”^2^. So too is the precarious fate, and need for conservation, of *D. lindenii*.

## Data Availability

Images supporting the results are archived with National Geographic and the Audubon Society.
